# Pax7 Is Necessary and Sufficient for the Myogenic Specification of CD45^+^:Sca1^+^ Stem Cells from Injured Muscle

**DOI:** 10.1371/journal.pbio.0020130

**Published:** 2004-05-11

**Authors:** Patrick Seale, Jeff Ishibashi, Anthony Scimè, Michael A Rudnicki

**Affiliations:** **1**Department of Biology, McMaster UniversityHamilton, OntarioCanada; **2**Ottawa Health Research Institute, Molecular Medicine ProgramOttawa, OntarioCanada

## Abstract

CD45^+^:Sca1^+^ adult stem cells isolated from uninjured muscle do not display any myogenic potential, whereas those isolated from regenerating muscle give rise to myoblasts expressing the paired-box transcription factor Pax7 and the bHLH factors Myf5 and MyoD. By contrast, CD45^+^:Sca1^+^ isolated from injured *Pax7* **^−/−^** muscle were incapable of forming myoblasts. Infection of CD45^+^:Sca1^+^ cells from uninjured muscle with retrovirus expressing Pax7 efficiently activated the myogenic program. The resulting myoblasts expressed Myf5 and MyoD and differentiated into myotubes that expressed myogenin and myosin heavy chain. Infection of CD45**^−^**:Sca1**^−^** cells from *Pax7* **^−/−^** muscle similarly gave rise to myoblasts. Notably, infection of *Pax7*-deficient muscle with adenoviral Pax7 resulted in the de novo formation of regenerated myofibers. Taken together, these results indicate that Pax7 is necessary and sufficient to induce the myogenic specification of CD45^+^ stem cells resident in adult skeletal muscle. Moreover, these experiments suggest that viral transduction of Pax7 is a potential therapeutic approach for the treatment of neuromuscular degenerative diseases.

## Abstract 2

The gene Pax7 is shown to be a key regulator of muscle cell differentiation in specific populations of adult stem cells during muscle tissue regeneration

## Introduction

Skeletal muscle regeneration has long been considered to be mediated solely by monopotential skeletal muscle stem cells known as satellite cells (Bischoff 1994; Charge and Rudnicki 2004). However, recent studies have identified novel populations of adult stem cells in skeletal muscle. For example, “side-population” (SP) cells isolated from muscle tissue participate in the regeneration of skeletal muscle and give rise to satellite cells (Gussoni et al. 1999; Asakura et al. 2002). In vitro, muscle SP cells readily form hematopoietic colonies, but do not spontaneously differentiate into muscle cells unless cocultured with satellite-cell-derived myoblasts (Asakura et al. 2002).

Various cell surface markers have been employed to purify adult stem cell populations from skeletal muscle, including c-kit, Sca1, CD34, and CD45 (reviewed by Charge and Rudnicki 2004). Almost all muscle-derived hematopoietic progenitor and blood reconstitution activity is derived from CD45^+^ cells (Asakura et al. 2002; McKinney-Freeman et al. 2002). Muscle-derived CD45^+^ cells purified from uninjured muscle are uniformly nonmyogenic in vitro and do not form muscle in vivo (Asakura et al. 2002; McKinney-Freeman et al. 2002). However, coculture and in vivo injection experiments indicate that CD45^+^ SP, as well as CD45^−^ SP, cells possess myogenic potential (Asakura et al. 2002; McKinney-Freeman et al. 2002).

Recent experiments have established that CD45^+^ adult stem cells have a normal physiological role in tissue regeneration (Polesskaya et al. 2003). CD45^+^:Sca1^+^ cells display a 30-fold expansion in number following cardiotoxin-induced (ctx-induced) injury. Importantly, a large proportion of CD45^+^:Sca1^+^ cells isolated from regenerating muscle acquire myogenic potential and appear to represent a significant source of myogenic progenitors during regenerative myogenesis (Polesskaya et al. 2003). Moreover, the myogenic specification of these adult stem cells during regeneration occurs by a Wnt-signaling-dependent mechanism (Polesskaya et al. 2003).

The paired-box transcription factor Pax7 is specifically expressed in satellite cells and is required for the specification of the satellite cell lineage (Seale et al. 2000). Following Wnt treatment of isolated CD45^+^ adult stem cells, Pax7 is rapidly induced as an early marker of satellite cell myogenic specification (Polesskaya et al. 2003). Together, these data suggest the hypothesis that Pax7 represents the target of Wnt signaling that directs the myogenic specification of adult stem cells resident in muscle. To investigate this hypothesis, we examined the myogenic potential of adult stem cells from *Pax7* **^−/−^** muscle, and employed viral vectors to transduce Pax7 into cells in vivo and in vitro. Our experiments demonstrate that Pax7 induces the myogenic program in specific populations of adult stem cells within muscle tissue and support the conclusion that Pax7 regulates myogenic determination during regenerative myogenesis.

## Results

### Pax7 Is Required for the Myogenic Commitment of CD45^+^:Sca1^+^ Cells

**Figure 1:**
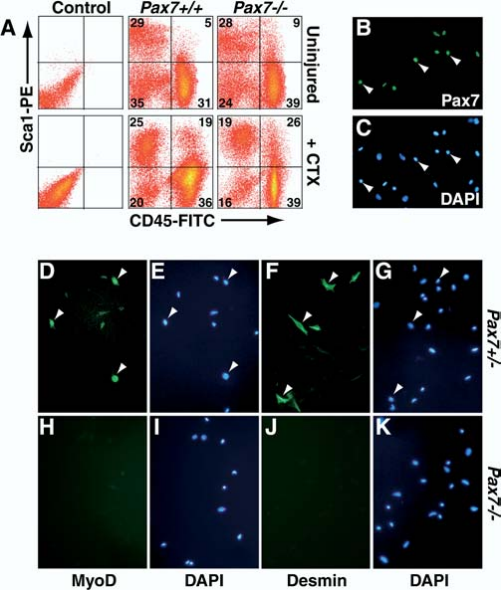
Pax7 Is Required for the Myogenic Specification of CD45^+^:Sca1^+^ Cells (A) Flow cytometric analysis of cell suspensions derived from uninjured and regenerating wild-type and *Pax7* **^−/−^** muscle (4 d after ctx injection) showed an increased proportion of CD45^+^ cells in *Pax7* **^−/−^** samples. (B and C) Pax7 protein was expressed in approximately 6%–10% of CD45^+^:Sca1^+^ cells purified from regenerating *Pax7^ +/−^* muscle. (D–K) MyoD (D and E) and Desmin (F and G) were induced in CD45^+^:Sca1^+^ cells from regenerating *Pax7^ +/−^* but were not expressed in CD45^+^:Sca1^+^ cells from regenerating *Pax7* **^−/−^** muscle (H–K).

To determine whether Pax7 is required for myogenesis in muscle-derived CD45^+^ cells, we analyzed the myogenic differentiation capacity of CD45^+^:Sca1^+^ cells from *Pax7* **^−/−^** muscle undergoing ctx-induced regeneration. Flow cytometry analysis revealed a higher average proportion of CD45-expressing cells in *Pax7* **^−/−^** muscle relative to wild-type (Figure 1A). Specifically, in muscle suspensions from *Pax7* **^−/−^** and wild-type littermates, 39% ± 4% versus 31% ± 9% of cells were CD45^+^:Sca1^−^ and 9% ± 2% versus 5% ± 2% of cells were CD45^+^:Sca1^+^, respectively (*n* ≥ 6). Four days following ctx injury, a significantly higher proportion of CD45^+^:Sca1^+^ cells (26% ± 3% compared to 19% ± 4% for *Pax7* **^−/−^** and wild-type, respectively, *p* < 0.05) and a reduced proportion of CD45^−^:Sca1^+^ cells were observed in *Pax7* **^−/−^** muscle (19% ± 4% compared to 25% ± 6% for *Pax7* **^−/−^** and wild-type, respectively, *p* = 0.07, *n* = 3) (Figure 1A).

By immunohistochemical analysis, Pax7 protein was upregulated in 6%–10% of CD45^+^:Sca1^+^ cells from wild-type muscle 4 d after ctx injury (Figure 1B and 1C). Importantly, Pax7 expression was not detected in CD45^+^:Sca1^+^ cells purified from uninjured muscles (Polesskaya et al. 2003). Furthermore, MyoD− (Figure 1D and 1E) and Desmin− immunoreactive cells (Figure 1F and 1G) were readily detected in cultured (18 h in growth medium) CD45^+^:Sca1^+^ cells purified from regenerating *Pax7^ +/^* **^−^** muscle (4 d post-ctx). By contrast, *Pax7* **^−/−^** CD45^+^:Sca1^+^ cells from regenerating muscle did not give rise to any MyoD-expressing (Figure 1H and 1I) or Desmin-expressing (Figure 1J and 1K) myogenic cells (*n* = 3 independent isolations with three mice per experiment). Taken together, these results support a central role for Pax7 in the myogenic specification of CD45^+^:Sca1^+^ cells in response to acute muscle damage.

### Pax7 Is Sufficient to Induce Myogenesis in CD45^+^:Sca1^+^ Cells

**Figure 2:**
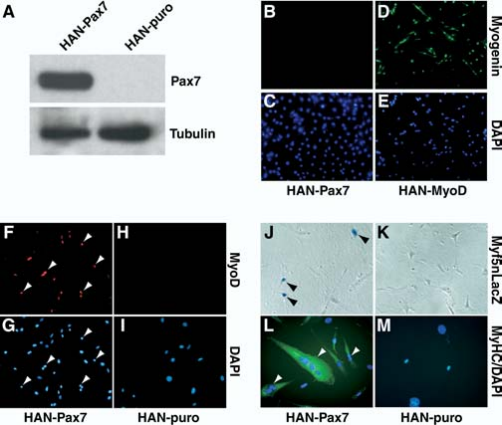
Pax7 Induces Myogenic Commitment in CD45^+^:Sca1^+^ Cells (A) Western blot analysis with anti-Pax7 antibody confirmed high levels of ectopic Pax7 in C3H10T1/2 cells infected with retrovirus-Pax7 (HAN-Pax7) but not with control virus expressing a puromycin-resistance marker (HAN-puro). (B and C) HAN-Pax7 did not induce expression of myogenin in C3H10T1/2 cells. (D and E) By contrast, MyoD virus (HAN-MyoD) efficiently converted C3H10T1/2 cells to myogenin-expressing myocytes (green) . (F–I) HAN-Pax7 (F and G) but not HAN-puro (H and I) activated expression of MyoD (red) in CD45^+^:Sca1^+^ cells from uninjured muscle. (J–M) HAN-Pax7 (J) but not HAN-puro (K) also induced Myf5nLacZ expression in CD45^+^:Sca1^+^ cells. Furthermore, HAN-Pax7-infected CD45^+^:Sca1^+^ cultures differentiated into MyHC-expressing myocytes (green) under differentiation conditions (L), whereas HAN-puro-infected cells did not undergo myogenic differentiation (M). DAPI staining (blue) was used to visualize all nuclei.

Adenoviral and retroviral expression systems were developed to ectopically introduce the Pax7 gene into putative adult stem cell populations. Pax7 was efficiently expressed from retrovirus (HAN-Pax7) in C3H10T1/2 fibroblasts and other cell cultures (Figure 2A). Stable expression of Pax7 did not induce MyoD (data not shown) or Myogenin protein expression (Figure 2B and 2C) in C3H10T1/2 cells. MyoD, as expected, readily converted C3H10T1/2 cells into skeletal myocytes (Figure 2D and 2E). These results show that Pax7 is not sufficient to induce myogenic determination in an established multipotent mesenchymal cell.

To determine whether Pax7 expression was sufficient to activate myogenesis in adult CD45^+^ progenitors, cells were fractionated from uninjured muscle and infected with Pax7-expressing retrovirus. Strikingly, CD45^+^:Sca1^+^ cells expressed Myf5 (data not shown) and MyoD (Figure 2F–2I) protein after infection only with Pax7 (HAN-Pax7), and not with puromycin-alone control virus (HAN-puro), indicating that these cells undergo myogenesis in response to Pax7

Infection of CD45^+^:Sca1^+^ cells from *Myf5nLacZ* reporter mice with HAN-Pax7 retrovirus specifically induced Myf5nLacZ expression and myogenesis in about 50% of infected cells (Figure 2J). The *Myf5nLacZ* allele faithfully recapitulates the expression pattern of the endogenous *Myf5* gene and is rapidly induced following myogenic commitment (Tajbakhsh et al. 1996; Tajbakhsh et al. 1997). Importantly, infection of CD45^+^:Sca1^+^ cells with control retrovirus expressing the puromycin-resistance gene (HAN-puro) did not activate Myf5nLacZ expression (Figure 2K). Moreover, exposure of these cultures to differentiation conditions caused Pax7-expressing cells to differentiate into myotubes expressing myosin heavy chain (MyHC) (Figure 2L and 2M). Ectopic expression of Pax7 in CD45^−^:Sca1^+^ or CD45^+^:Sca1^−^ cells did not result in the generation of myogenic cells. Taken together, these results demonstrate that Pax7 induces the myogenic program selectively in CD45^+^:Sca1^+^ adult stem cells from skeletal muscle.

### Expression of Pax7 Converted CD45^+^:Sca1^+^ Cells into Myogenic Progenitors

**Figure 3:**
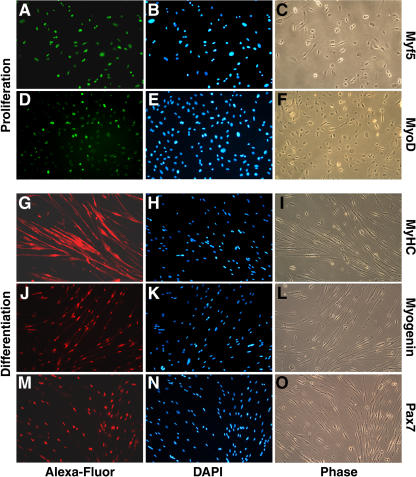
CDSC-Pax7 Cells Become Myogenic Progenitors Myf5 (A–C) and MyoD (D–F) protein (green) are expressed in proliferating CDSC-Pax7 cells. Exposure of CD45^+^:Sca1^+^ cultures to low mitogen medium induced the formation of multinucleated myotubes and expression of myogenic differentiation markers including MyHC (red) (G–I) and myogenin (red) (J–L). Sustained expression of Pax7 (red) (M–O) in these cultures did not interfere with their differentiation. DAPI staining (blue) was used to visualize all nuclei.

CD45^+^:Sca1^+^ cells expressing retroviral Pax7 were stably selected using puromycin, hereafter called CDSC-Pax7 cells (*n* = 4 independent isolates analyzed). CDSC-Pax7 cells displayed a stellate, fibroblastic morphology that was distinct from the round, refractile appearance of primary satellite-cell-derived myoblasts. Proliferating CDSC-Pax7 cells expressed the myogenic determination bHLH factors, Myf5 (Figure 3A–3C), and MyoD (Figure 3D–3F). CDSC-Pax7 cells cycled approximately three times faster than satellite-cell-derived myoblasts isolated simultaneously (data not shown) and maintained their myogenic identity as primary cultures in excess of three months. CDSC-Pax7 cultures also differentiated efficiently into multinucleated myotubes expressing the terminal differentiation markers MyHC (Figure 3G–3I) and myogenin (Figure 3J–3L). These results demonstrate that the constitutive expression of Pax7 (Figure 3M–3O), which is normally downregulated during differentiation (Seale et al. 2000), did not interfere with cell-cycle arrest and normal myotube formation. By contrast, overexpression of Pax7 in C2C12 myoblasts prevented their differentiation into MyHC-positive myocytes (data not shown). These experiments therefore demonstrate that myoblasts derived from Pax7-infected CD45^+^:Sca1^+^ stem cells are amenable to ex vivo expansion and subsequent terminal muscle differentiation.

### CDSC-Pax7 Cells Express High Levels of Myf5 and Sca1

**Figure 4:**
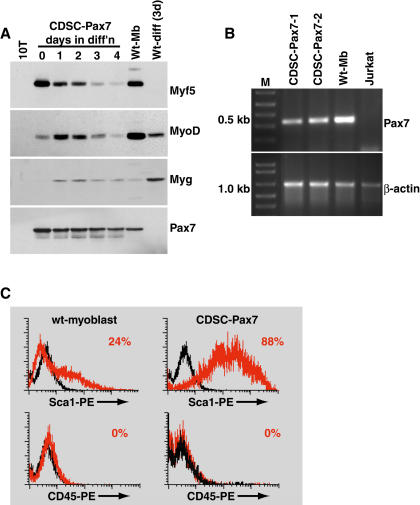
CDSC-Pax7 Cells Express High Levels of Myf5 and Sca1 (A) Western blot analysis of CDSC-Pax7 cells in proliferation conditions (day 0) and during differentiation (days 1–4) revealed high levels of Myf5 expression and low levels of MyoD expression. By contrast, satellite-cell-derived myoblasts (Wt-Mb) displayed the opposite profile of Myf5 and MyoD expression. Myogenin (Myg) was upregulated during the differentiation of CDSC-Pax7 and satellite-cell-derived myoblasts (Wt-diff). Note the sustained expression of Pax7 during the differentiation of CDSC-Pax7 cells. C3H10T1/2 (10T) lysate was used as a negative control. (B) RT-PCR analysis indicated that CDSC-Pax7 cells (two different lines) upregulated the endogenous *Pax7* mRNA. Satellite-cell-derived myoblasts (Wt-Mb) and Jurkat cells were used as positive and negative controls, respectively. (C) Flow cytometry indicated that CDSC-Pax7 cells lost expression of CD45 but retained high levels of Sca1. About 24% of satellite-cell-derived myoblasts (wt-myoblasts) expressed low levels of Sca1. (Black graph depicts staining with IgG-PE control antibody; red graph shows target staining using Sca1-PE or CD45-PE.)

The expression pattern of myogenic factors in proliferating and differentiating CDSC-Pax7 cell lines was analyzed by Western blot (*n* = 2). These experiments indicated that Myf5 was expressed at high levels in proliferating CDSC-Pax7 cells (Figure 4A; day 0). Moreover, CDSC-Pax7 cells continued to express Myf5 protein during their differentiation. CDSC-Pax7 cells also expressed MyoD but at low levels relative to primary myoblasts. MyoD was transiently upregulated in CDSC-Pax7 cells as they entered their differentiation program (Figure 4A; days 1 and 2).

The primary myogenic regulatory factor (MRF) expression profile in CDSC-Pax7 cells contrasted with the pattern observed in satellite-cell-derived primary myoblasts (Figure 4; Wt-Mb). Primary myoblasts expressed higher levels of MyoD and lower levels of Myf5 and downregulated Myf5 immediately upon differentiation (Wt-diff). Myogenin (Myg) was upregulated during the differentiation of CDSC-Pax7 cells, albeit at lower levels compared with differentiating satellite-cell-derived myoblasts (Wt-diff). Interestingly, CDSC-Pax7 cells also expressed endogenous *Pax7* mRNA as demonstrated by reverse transcriptase PCR (RT-PCR) using primers that amplify a sequence within the *Pax7* 3′ UTR that is not present in the viral-Pax7 vector (Figure 4B). This result suggests that autoregulatory mechanisms may control *Pax7* gene expression. Taken together, these analyses demonstrate that CDSC-Pax7 cells and primary satellite-cell-derived myoblasts express different levels of MyoD and Myf5 but are similar in their ability to undergo terminal differentiation.

CDSC-Pax7 cells were originally derived from cells expressing cell surface CD45 and Sca1 proteins. Flow cytometry was employed to determine whether expression of these markers was maintained in vitro. Notably, CDSC-Pax7 cells continued to express high levels of Sca1 (approximately 90% of cells showed intense staining), but CD45 expression was extinguished (Figure 4C). Interestingly, approximately 24% of primary satellite-cell-derived myoblasts displayed low levels of Sca1 staining. Sca1 levels were not increased in satellite-cell-derived myoblasts overexpressing Pax7, demonstrating that CDSC-Pax7 cells did not arise from a small number of committed myoblasts fractionated with CD45^+^:Sca1^+^ cells (data not shown).

### CDSC-Pax7 Cells Differentiate In Vivo

**Figure 5:**
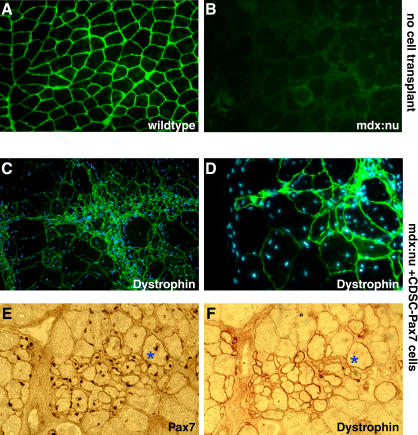
CDSC-Pax7 Cells Efficiently Contribute to the Repair of Dystrophic Muscle (A) Wild-type muscle expressed dystrophin at the plasmalemma of all myofibers. (B) Dystrophin protein was not detected in muscle sections from *dystrophin*-deficient *mdx:nude* mice *(mdx:nu).* (C–F) CDSC-Pax7 cells differentiated in vivo after transplantation, readily forming large numbers of dystrophin-expressing myofibers (green) in *mdx:nude* muscle (C and D). Serial cross sections showing the viral expression of Pax7 protein in central nuclei of regenerated fibers (red staining in [E]) confirmed the donor origin of dystrophin-positive myofibers (red staining in [F]).

To establish whether CDSC-Pax7 cells were capable of integrating and differentiating as myofibers in vivo, intramuscular transplantation studies were performed in dystrophic (*dystrophin*-deficient) muscle. Specifically, 1 × 10^5^ CDSC-Pax7 cells were injected into the tibialis anterior (TA) muscle of 4- to 6-week-old *mdx:nude* mice. *Mdx* mice carry a point mutation in the *dystophin* gene and are a mouse model of Duchenne muscular dystrophy (Bulfield et al. 1984; Sicinski et al. 1989; Blaveri et al. 1999). As expected, dystrophin was localized at the myofiber sarcolemma in wild-type muscle (Figure 5A) and was absent in *mdx:nude* skeletal muscle (Figure 5B). Two months after transplantation, TA muscles were processed for immunohistochemical detection of dystrophin and Pax7. These experiments revealed that CDSC-Pax7 cells differentiated in vivo, readily forming dystrophin-expressing myofibers in the *dystrophin*-deficient recipient muscle (Figure 5C and 5D). Endogenous Pax7 protein expression was not observed in central nuclei within differentiated wild-type myofibers (data not shown). Therefore, the expression of Pax7 protein (from retrovirus) in central nuclei within dystrophin^+^ fibers established the contribution of CDSC-Pax7 donor cells to recipient muscles (Figure 5E and 5F). These results thus document the capacity for CDSC-Pax7 cells to differentiate in vivo and contribute to the repair of dystrophic muscle.

### Pax7 Does Not Induce Myogenesis in CD45^+^:Sca1^+^ Cells from *Pax7* ^−/−^ Muscle

**Figure 6:**
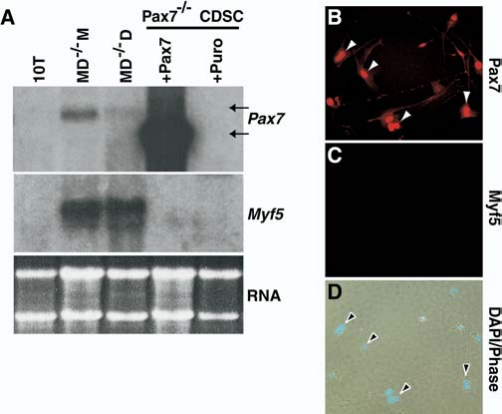
Pax7 Does Not Induce Myogenesis in CD45^+^:Sca1^+^ Cells from *Pax7* **^−/−^** Muscle (A) Northern analysis shows that *MyoD* **^−/−^** satellite-cell-derived myoblasts (*MD* **^−/−^** M) and differentiating cells (*MD* **^−/−^** D) express endogenous *Pax7* (upper arrow, Pax7 blot) and *Myf5* transcripts. *Pax7* **^−/−^** CD45^+^:Sca1^+^ cells (CDSC) transduced with HAN-Pax7 (+Pax7) or HAN-puro (+puro) did not initiate expression of *Myf5* mRNA. The retroviral transcript producing Pax7 (lower arrow) is smaller than the endogenous *Pax7* mRNA (e.g., lower arrow). (B–D) Ectopic expression of Pax7 (red) (B) in *Pax7* **^−/−^** CDSC cells did not induce Myf5 protein expression (C). DAPI staining (blue) was used to visualize nuclei (D).

The myogenic differentiation of wild-type CD45^+^:Sca1^+^ muscle cells suggested that ectopic Pax7 would induce myogenesis in this cell population from *Pax7* **^−/−^** muscle. Infection of *Pax7* **^−/−^** CD45^+^:Sca1^+^ cells with Pax7 retrovirus resulted in high levels of retroviral Pax7 transcript but no expression of *Myf5* mRNA by Northern blot hybridization (Figure 6A) or RT-PCR (data not shown). The absence of Myf5 (Figure 6B–6D) or MyoD (data not shown) expression, determined by immunochemical staining of Pax7-transduced cells, ruled out the possibility that a minor subpopulation of CD45^+^:Sca1^+^ cells underwent myogenesis. These experiments illustrate that *Pax7* **^−/−^** CD45^+^:Sca1^+^ cells do not enter the myogenic lineage in response to Pax7, suggesting that intrinsic differences exist between wild-type and *Pax7*-deficient populations of CD45^+^:Sca1^+^ cells.

### Pax7 Promotes Myogenic Commitment in *Pax7*-Deficient CD45^−-^:Sca1^−^ Cells

**Figure 7:**
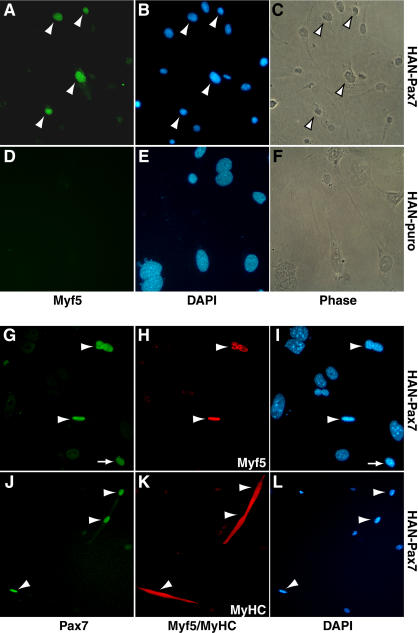
Pax7 Promotes Myogenesis in CD45^−^:Sca1^−^ Cells from *Pax7* **^−/−^** Muscle (A–C) Ectopic expression of Pax7 (HAN-Pax7) induced Myf5 expression (green) and myogenic commitment in CD45^−^:Sca1^−^ cells from *Pax7* **^−/−^** muscle. (D–F) By contrast, Myf5-expressing cells were completely absent from HAN-puro-infected cultures after selection. (G–L) CD45^−^:Sca1^−^ cells from *Pax7* **^−/−^** muscle expressed Myf5 (red) (H) and MyHC (red) (K) only in cells that also coexpressed high levels of Pax7 protein (G and J). Arrowheads indicate cells coexpressing Pax7 and Myf5/MyHC. Arrow in (G) and (I) depicts a Pax7^+^, Myf5^−^ cell.

In cell suspensions from uninjured muscle, satellite cells and their daughter myogenic precursors are uniformly CD45^−^ and Sca1^−^ (Polesskaya et al. 2003). In *Pax7* **^−/−^** mice, the extremely rare myogenic cells in muscle tissue do not express CD45 or Sca1, and do not survive or expand in a variety of culture conditions (S.B.P. Chargé, P. Seale, and M.A. Rudnicki, unpublished data). Interestingly, ectopic expression of Pax7 in CD45^−^:Sca1^−^ cells isolated from *Pax7* **^−/−^** muscle resulted in the expression of Myf5 protein in more than 50% of infected cells (*n* = 3) (Figure 7A–7C). Analysis of HAN-puro-infected control cultures did not reveal any myogenic cells (Figure 7D–7F). Importantly, all Myf5-expressing myoblasts (Figure 7G–7I) and MyHC-expressing differentiated myotubes (Figure 7J–7L) in Pax7-infected CD45^−^:Sca1^−^ cultures expressed viral Pax7.

In these experiments we cannot formally exclude the possibility that Pax7 promoted the survival and proliferation of committed myoblasts. However, given the extremely low number of myogenic cells recovered in culture (less than 0.7%), the low efficiency of primary myoblast infection (approximately 5%–10%), and the absence of any Myf5- or MyoD-expressing cells in control HAN-puro cultures, our results strongly suggest that Pax7 induces myogenic specification in a nonmyoblast, CD45- and Sca1-negative cell.

### Adenoviral Expression of Pax7 Enhances Regeneration in *Pax7*-Deficient Muscle

To investigate whether Pax7 was sufficient to stimulate myogenesis in vivo, adenovirus was used to ectopically express Pax7 in damaged *Pax7* **^−/−^** muscle. Adenoviral particles (1 × 10^8^) expressing either Pax7 (Ad-Pax7) or the bacterial *β-galactosidase* gene *(LacZ)* (Ad-LacZ) were injected directly into injured TA muscles of 4- to 6-week-old *Pax7* **^−/−^** animals 2 d after administration of ctx (*n* = 3). Immunohistochemistry for Pax7 in adenovirus-infected muscles demonstrated widespread Pax7 expression primarily in mononuclear cells within the damaged tissue (data not shown).

**Figure 8:**
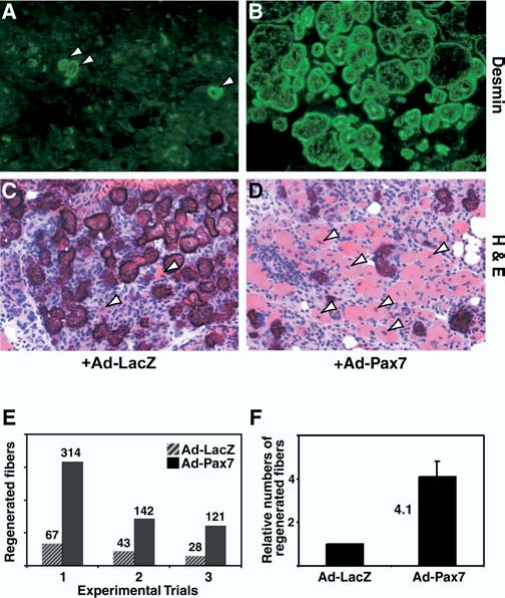
Adenovirus-Pax7 Significantly Improves Regeneration In Vivo (A and B) Infection of ctx-damaged *Pax7* **^−/−^** muscles with Ad-Pax7 resulted in markedly improved muscle integrity and a significantly increased number of Desmin immunoreactive (green) regenerated fibers (B) relative to muscles treated with Ad-LacZ (A). (C and D) Hematoxylin and Eosin staining similarly showed an increased number of centrally nucleated fibers in Ad-Pax7-treated *Pax7* **^−/−^** muscles. (E) In three separate experimental trials, the number of regenerated fibers was markedly increased in Ad-Pax7-treated muscles relative to Ad-LacZ; however, the response was biologically variable between groups. On average, Ad-Pax7 infection resulted in a 4.1 ± 0.72–fold increase in regenerated *Pax7* **^−/−^** myofibers (F).

To assess the effect of Pax7 expression in damaged tissue, TA muscles were analyzed and scored for regeneration 12 d after infection by enumerating the number of regenerated fibers with centrally located nuclei. The newly regenerated status of centrally nucleated fibers was confirmed by Desmin and embryonic MyHC immunoreactivity. Ad-Pax7 induced a markedly enhanced regenerative response relative to Ad-LacZ in *Pax7* **^−/−^** muscle as evidenced by the increased number of Desmin-positive (Figure 8A and 8B) and centrally nucleated fibers (Figure 8C and 8D).

Wild-type TA muscles typically contained in excess of 700 regenerated fibers 14 d after injury (data not shown). In three independent experiments, ctx-damaged TA muscle from *Pax7* **^−/−^** mice typically contained an average of 46 surviving or regenerated fibers following regeneration (Figure 8E). By contrast, infection of regenerating *Pax7* **^−/−^** TA with Ad-Pax7 resulted in the generation of an average of 192 myofibers (Figure 8E). Therefore, Pax7-infected tissue contained a 4.1 ± 0.72–fold increase in the number of regenerated fibers (Figure 8F). Together, these results demonstrate the ability of virally transduced Pax7 to direct the de novo generation of myogenic progenitors capable of forming new myofibers and participating in regenerative myogenesis.

## Discussion

In this article, we demonstrate that expression of Pax7 induces the myogenic specification of CD45^+^ muscle-derived adult stem cells. First, CD45^+^:Sca1^+^ cells isolated from regenerating *Pax7* **^−/−^** muscle were incapable of undergoing myogenic specification (see Figure 1). Second, expression of Pax7 with viral vectors in CD45^+^:Sca1^+^ cells purified from uninjured muscle promoted the formation of highly proliferative myoblasts that readily differentiated as multinucleated myotubes (see Figures 2 and 3). CD45^+^:Sca1^+^ cells engineered to express Pax7 (CDSC-Pax7) also differentiated in vivo, readily contributing to the regeneration of dystrophic muscle (see Figure 5). Lastly, Ad-Pax7 gene delivery into chemically damaged *Pax7* **^−/−^** muscle resulted in the efficient de novo generation of myofibers in the absence of endogenous satellite cells. Taken together, these data unequivocally establish that Pax7 plays a key regulatory role for directing myogenic specification in some populations of adult stem cells during regenerative myogenesis. Moreover, these results emphasize the possibility of designing strategies to upregulate or ectopically express Pax7 in stem cells for the treatment of muscle degenerative diseases.

The presence of adult stem cell populations distinct from satellite cells resident in skeletal muscle tissue has been well documented (Gussoni et al. 1999; Jackson et al. 1999; Torrente et al. 2001; Asakura et al. 2002; McKinney-Freeman et al. 2002; Qu-Petersen et al. 2002; Cao et al. 2003). An understanding of the developmental origin of these various cell populations and their physiological relevance in the maintenance of tissue integrity is beginning to emerge. Several lines of evidence argue that skeletal muscle regeneration is normally mediated entirely by stem cells resident in muscle tissue. First, destruction of stem cells resident in muscle with high-dose local irradiation of limbs results in a long-term deficit in muscle growth and regeneration (Wakeford et al. 1991; Pagel and Partridge 1999; Heslop et al. 2000). Second, transplanted muscles do not incorporate host nuclei after injury and regeneration (Schultz et al. 1986). Together, those experiments argue that CD45^+^ stem cells from marrow do not normally transit in significant numbers through the circulation to sites of muscle damage. Our experiments, however, suggest that a population of specialized CD45^+^ cells resides in muscle and can efficiently form myogenic progenitors in response to Wnt signaling (Polesskaya et al. 2003). In the current work we demonstrate that induction of Pax7 is required for the myogenic specification of CD45^+^ stem cells and that retroviral transduction can dominantly induce the myogenic specification of these cells. These observations therefore provide compelling evidence that some adult stem cells participate in regenerative myogenesis by forming myogenic progenitors following Pax7 induction in response to Wnt signaling. These data additionally suggest the hypothesis that Pax7 is a transcriptional target of the β-Catenin complex in Wnt-stimulated adult stem cells.

Interesting parallels exist between the inductive mechanisms and transcriptional networks in embryonic and regenerative myogenesis (Parker et al. 2003). For example, the Pax7-dependent myogenic specification of CD45^+^ adult stem cells appears analogous to the Pax3-dependent induction of muscle precursors during somitogenesis. In the early embryo, Pax3 is expressed in the presomitic mesoderm and immature epithelial somites prior to the onset of muscle-specific gene expression (Goulding et al. 1994; Williams and Ordahl 1994). Moreover, Pax3 functions upstream of MyoD in the formation of trunk and body-wall muscle (Tajbakhsh et al. 1997). Consistent with a direct role for Pax3 in myogenic induction, ectopic Pax3 activates MyoD expression in embryonic tissues (Maroto et al. 1997; Bendall et al. 1999; Heanue et al. 1999). However, Pax3 also regulates cell survival in the presomitic mesoderm in areas that do not express Pax7, suggesting an indirect mechanism by which Pax3 may act genetically upstream of MyoD (Borycki et al. 1999). Our experiments do not rule out the possibility that Pax7 promotes the survival of CD45^+^ progenitors that are already competent to give rise to myogenic cells. Characterization of the downstream targets of Pax7 in CD45^+^ cells will be required to directly address this issue.

In explanted embryonic tissues, signals from the floor plate and neural tube are required for induction of the MRFs (Munsterberg and Lassar 1995; Pourquie et al. 1995, 1996; Cossu et al. 1996). In particular, Wnt7a activates expression of MyoD in explanted paraxial mesoderm from 10.5-d-old mouse embryos (Tajbakhsh et al. 1998). The requirement for Pax3 expression in somitic precursors prior to the onset of MyoD expression suggests that Wnt signals may activate Pax3 and indirectly promote MRF expression (Borycki et al. 1999). An analogous requirement for Pax7 in the myogenic commitment of adult CD45^+^ progenitors suggests a conserved hierarchy whereby Wnt signaling activates Pax3 or Pax7 expression upstream of the MRFs in somitic and adult muscle stem cells, respectively. This notion is supported by the observed loss of Pax3 expression in P19 mesodermal precursors engineered to express a dominant negative form of the Wnt effector protein, β-Catenin (Petropoulos and Skerjanc 2002).

A confounding result from our study was the inability of Pax7 to induce myogenesis in CD45^+^:Sca1^+^ cells recovered from *Pax7* **^−/−^** muscle (see Figure 6). Several possible explanations may account for this observation. First, CD45^+^:Sca1^+^ muscle cells represent a heterogeneous cell population, as evidenced by their nonuniform response to stimuli such as myoblast coculture, Wnt proteins, and ectopic expression of Pax7 (results herein and Polesskaya et al. 2003). Analysis of muscle suspensions from young *Pax7* **^−/−^** mice revealed a significantly increased number of hematopoietic progenitors and adipogenic cells (Seale et al. 2000). We also observed altered proportions of CD45- and Sca1-expressing cells in uninjured and regenerating muscle (see Figure 1A). The putative stem cell subfraction coexpressing CD45 and Sca1 may have been exhausted prematurely during postnatal *Pax7* **^−/−^** muscle development. It is also conceivable that a reduced proportion of stem cells in the *Pax7* **^−/−^** CD45^+^:Sca1^+^ muscle fractions was not detected in our assay due to a low efficiency of retroviral transduction (approximately 10% of surviving CD45^+^:Sca1^+^ cells with GFP virus). The identification of additional markers expressed by adult muscle-derived stem cells is required to more thoroughly explore these issues.

Alternatively, adult stem cells may require additional signals to undergo myogenesis in response to Pax7. The profound growth deficit in *Pax7* **^−/−^** muscles is likely to invoke nonspecific and indirect changes to the muscle microenvironment (Seale et al. 2000). Specific cues required to “prime” or activate adult stem cells may thus be absent or ineffective in *Pax7* **^−/−^** muscle. Finally, our experiments also revealed that the endogenous *Pax7* gene is upregulated during the myogenic specification of CD45^+^:Sca1^+^ cells (Figure 4B). Therefore, endogenous gene activity, possibly through the regulated expression of different isoforms (Kay et al. 1995; Ziman et al. 1997), may be essential to the stability of myogenic commitment. Future experiments addressing the functional differences between CD45^+^:Sca1^+^ cells in wild-type and *Pax7*-deficient muscle will provide a unique opportunity to gain a more complete understanding of the role of these cells during postnatal muscle development.

Although CD45^+^ cells from *Pax7* **^−/−^** muscle were apparently unable to undergo myogenesis, ectopic Pax7 induced expression of Myf5 and myogenic specification in *Pax7*-deficient CD45^−^:Sca1^−^ cells (see Figure 7). Moreover, Ad-Pax7 significantly increased the in vivo regenerative capacity of *Pax7* **^−/−^** muscle (see Figure 8). Skeletal muscle in adult *Pax7* **^−/−^** mice displays a profound regeneration deficit with only occasional regenerated fibers observed at the site of injury 30 d after ctx injection (S.B.P. Chargé, P. Seale, and M.A. Rudnicki, unpublished data). Taken together, these results imply the presence of *Pax7* **^−/−^** muscle progenitors that require the activity of Pax7 to generate sufficient numbers of myoblasts for effective regeneration. Further studies will be required to molecularly characterize the responsive cells and their developmental relationship to other muscle stem cell populations.

The dominant expression of Myf5 in Pax7-infected CD45^+^:Sca1^+^ cells (CDSC-Pax7) (see Figure 4A) suggests a paradigm wherein Pax7 preferentially activates Myf5 compared to MyoD. Interestingly, Pax3 has been implicated in myogenesis specifically upstream of MyoD (Tajbakhsh et al. 1997). Taken together, these observations suggest the hypothesis that Pax3 and Pax7 specify distinct myogenic lineages through the preferential activation of MyoD and Myf5, respectively.

Several experimental observations have noted a role for Myf5 in promoting myoblast proliferation. For example homozygous *Myf5nLacZ*, (e.g., *Myf5*-deficient) embryos display significantly reduced numbers of LacZ-expressing myogenic progenitors (Tajbakhsh et al. 1996). In avian embryos, Myf5 is preferentially expressed in proliferating myoblasts, whereas MyoD appears to be upregulated in differentiating cells (Delfini et al. 2000). Furthermore, *Myf5^ −/−^* satellite-cell-derived myoblasts display a profound proliferation deficit (Montarras et al. 2000). The increased growth rate of CDSC-Pax7 cells is reminiscent of *MyoD^−/−^* myoblasts that also express elevated levels of Myf5 (Sabourin et al. 1999). These observations raise the possibility that Pax7 activates expression of Myf5 to promote adult myoblast expansion whereas Pax3 preferentially induces MyoD and differentiation.

The requirement for Pax7 in the specification of muscle satellite cells (Seale et al. 2000) and its induction during the myogenic recruitment of CD45^+^ adult stem cells provide further evidence for a developmental relationship between CD45^+^ adult muscle stem cells and satellite cells. Together, our experiments suggest the hypothesis that CD45^+^:Sca1^+^ cells give rise to satellite cells by a Pax7-dependent mechanism in response to Wnt signals. In conclusion, our work establishes that Pax7 is necessary and sufficient for the myogenic specification of specific populations of adult stem cells resident in muscle tissue. The proliferative myogenic character of CDSC-Pax7 cells and their efficient engraftment into dystrophic muscle further argue that methods to deliver Pax7 or upregulate its expression in stem cells may be useful in treating degenerative muscle disease.

## Materials and Methods

### 

#### Mice

Mice carrying a targeted null mutation in *Pax7* (hereafter referred to as *Pax7^ −/−^*) were generously provided by Drs. A. Mansouri and P. Gruss (Mansouri et al. 1996) and outbred into the SV129 background to increase survival. *Myf5nLacZ* mice were provided by Dr. S. Tajbakhsh (Tajbakhsh et al. 1996). *Mdx* mice were obtained from Jackson Laboratory (Bar Harbor, Maine, United States). *Mdx:nu* mice were provided by Dr. T.A. Partridge (see Blaveri et al. 1999).

#### Cell sorting

Mononuclear cells were recovered from uninjured hindlimb muscles or from ctx-damaged TA muscles of *Pax7^ +/+^*,*Pax7^ +/−^*, and *Pax7* **^−/−^** mice as described previously (Megeney et al. 1996). Cells were washed twice with ice-cold DMEM supplemented with 5% FBS, passed through 30-μm filters (Miltenyi Biotec, Bergisch Gladbach, Germany) and suspended at a concentration of 2–3 × 10^6^ cells/ml. Staining was performed for 30 min on ice using the antibodies CD45-APC (30-F11), CD45.2-FITC (104), Sca1-PE, or FITC (D7), all from BD Biosciences Pharmingen (San Diego, California, United States) and CD45-TC (30-F11) from Caltag Laboratories (Burlingame, California, United States). Primary antibodies were diluted in cell suspensions at 1:200. After two washes with cold PBS supplemented with 2% FBS, cells were separated on a MoFlo cytometer (DakoCytomation, Glostrup, Denmark). Sort gates were strictly defined based on isotype control stained cells and single antibody staining. Dead cells and debris were excluded by gating on forward and side scatter profiles. Sorting was performed using single cell mode to achieve the highest possible purity. The purity of sorted populations was routinely greater than 98%.

#### Retroviral and adenoviral gene expression

Retrovirus was produced according to the 3-plasmid HIT system with plasmids pHIT60, pHIT456, and pHAN-puro as described elsewhere (Soneoka et al. 1995). pHIT60 encodes the MLV retroviral gag-pol, pHIT456 expresses an amphotrophic envelope protein, and pHAN-puro is an expression vector with a hybrid CMV-5′ LTR promoter driving production of the retroviral transcript. Pax7 expression vectors were generated using the mouse Pax7d isoform containing a single Ala→Thr substitution at the seventh amino acid (the Thr residue is conserved in human, chicken, and zebrafish Pax7 proteins). Pax7d or mouse MyoD are translated from the full retroviral transcript, whereas the puromycin-resistance marker is expressed following integration from a shorter transcript produced by the SV40 early promoter located 3′ to the multiple cloning site. Transient cotransfection of all three plasmids into 293FT cells (Invitrogen, Carlsbad, California, United States) by the calcium phosphate method (Graham and van der Eb 1973) routinely produced viral titres between 10^6^ and 10^7^ cfu per ml. pHAN-puro was used to produce puromycin-resistant virus for controls.

Purified CD45^+^:Sca1^+^ or CD45^−^:Sca1^−^ cells were spun down, counted, and 20,00–50,000 cells were then cultured overnight on collagen-coated 4-well chamber slides in HAM's F10 medium (Invitrogen) supplemented with 20% FBS, antibiotics, and 10 ng/ml Stem Cell Factor (R & D Systems, Minneapolis, Minnesota, United States). The following day, cells were incubated for 6 h with retrovirus at a 1:1 ratio (complete medium: retrovirus supernatant) with 8 μg/ml polybrene (hexadimethrine bromide; Sigma, St. Louis, Missouri, United States). After infection, cells were rinsed twice with PBS, and all cells were replated in myoblast growth medium. After 48 h, infected pools were selected in 1 μg/ml puromycin (Sigma) to establish stable CDSC-Pax7 lines. C3H10T1/2 cells were incubated overnight with MyoD, Pax7, or puro virus and 8 μg/ml polybrene.

Adenovirus (type V) was prepared using the Ad-Max adenovirus creation kit (Microbix Biosystems, Toronto, Ontario, Canada). Ad-Pax7d (cDNA as described above) and Ad-LacZ were expressed from the murine CMV promoter. Adenovirus was purified in CsCl gradients by centrifugation, dialyzed against sterile PBS, and frozen down in 15% glycerol at −80 °C. Titres of purified adenovirus were determined by plaque assays on 293 cells and were always above 10^10^ pfu/ml.

#### Western blot analyses

Cell cultures were lysed in RIPA extraction buffer (50mM Tris-HCl [pH 7.4], 1% Nonidet P-40, 0.5% NaDeoxycholate, 0.1% Sodium-dodecyl-sulphate, 5 mM EDTA, 150 mM NaCl, 50 mM NaF) supplemented with protease inhibitors (Complete; Roche, Basel, Switzerland). The extracts were normalized for protein content using Bio-Rad dye (Hercules, California, United States). Forty micrograms of lysate was separated by sodium-dodecyl-sulfate-polyacrylamide gel electrophoresis and transferred to PVDF filters (ImmobilonP; Millipore, Billerica, Massachusetts, United States). Filters were probed with antibodies to Pax7 (Developmental Studies Hybridoma Bank [DSHB], Iowa City, Iowa, United States); Myf5, 1:1000 (C-20, Santa Cruz Biotechnology, Santa Cruz, California, United States); MyoD, 1:1000 (C-20, Santa Cruz Biotechnology); myogenin (F5D, DSHB); and α-tubulin, 1:2000 (T 9026, Sigma). Secondary detection was performed with horseradish peroxidase-conjugated antibodies (Bio-Rad). Protein expression was visualized using the ECL Plus kit (Amersham Biosciences, Little Chalfont, United Kingdom).

#### Ctx-induced regeneration and in vivo adenovirus infections

Four- to six-week-old *Pax7* **^−/−^** and wild-type littermates were anesthetized with Halothane gas. Twenty-five microliters of 10 μM ctx (Latoxan, Valence, France) was injected into the midbelly of the TA muscle, using a 29½ G insulin syringe. Mice were sacrificed at 4 d or 2 wk after ctx injection. For adenovirus infections, 25 μl of sterile PBS containing 10^8^ particles of purified Ad-Pax7 or Ad-LacZ was injected 2 d after ctx injection into damaged TA muscles with a 29½ G insulin syringe.

#### Cell transplantation

Primary CDSC-Pax7 cells cultured in myoblast conditions were trypsinized, washed twice with PBS, and suspended at 5 × 10^5^ cells/25-μl in sterile PBS for cell transplantation. Cells were injected directly into the TA midbelly of 4- to 6-wk-old *mdx:nude* mice. Mice were sacrificed 2 mo after cell injections to analyze the myogenic contribution of transplanted cells.

#### Cell cultures

Primary satellite-cell-derived myoblasts were established from purified CD45^−^:Sca1^−^ fractions of hindlimb muscle of 4- to 6-wk-old *Pax7^ +/+^* or *Pax7^ +/−^* mice. Myoblasts and CDSC-Pax7 cells were maintained in HAM's F-10 medium (Invitrogen) supplemented with 20% FBS and 2.5 ng/ml bFGF (Invitrogen) on collagen-coated dishes. CDSC-Pax7 cells and primary satellite-cell-derived myoblasts were differentiated for 1–3 d in DMEM supplemented with 5% horse serum. C3H10T1/2 and HEK 293 cells were obtained from the ATCC (Manassas, Virginia, United States) and maintained in DMEM supplemented with 10% FBS.

#### Histology and immunocytochemistry

For analysis of regeneration and enumeration of regenerated myofibers, TA muscles were isolated, embedded in OCT (Tissue-Tek; Sakura Finetek, Torrance, California, United States)/20% sucrose and immediately frozen in liquid nitrogen. Ten-micrometer cryosections (cross sections) from the TA midbelly at the site of ctx injection were stained with Hematoxylin and Eosin. Central myonuclei in regenerating muscles were counted on at least two independent cross sections of the entire TA muscle per mouse analyzed. Fibers were further identified by immunostaining with antibodies specific to Desmin, 1:200 (D33, DakoCytomaton); dystrophin, 1:500 (Sigma); Pax7 (DSHB); or embryonic fast MyHC (F1.652, DSHB) followed by secondary detection with anti-mouse FITC conjugated antibody, 1:200 (Chemicon, Temecula, California, United States). Sections were analyzed on a Zeiss (Oberkochen, Germany) Axioplan 2 microscope.

Cultured cells were fixed with 4% paraformaldehyde, nonspecific antigens were blocked in 5% horse serum/PBS, and cells were reacted with primary antibodies as follows: Desmin, 1:200 (DakoCytomaton); MyoD, 1:200 (5.8A, BD Biosciences Pharmingen); all MyHC (MF-20, DSHB); Myf5, 1:1000 (C-20, Santa Cruz Biotechnology); Pax7 (DSHB); and myogenin (F5D, DSHB). Secondary detection was performed using fluorescein- or rhodamine-conjugated antibodies, 1:200 (Chemicon). *Myf5nLacZ* expression was detected by X-Gal reaction as described previously (Polesskaya et al. 2003).

#### RT-PCR and Northern blot analysis

Total RNA was extracted using RNeasy kits (Qiagen, Valencia, California, United States), according to manufacturer's instructions. RT-PCR analysis for endogenous *Pax7* mRNA was performed using the GeneAmp PCR Core kit (PerkinElmer, Wellesley, Massachusetts, United States). RT-PCR using 1 μg of total RNA was conducted as per manufacturer's instructions with the following modifications. cDNA synthesis was extended for 1 h at 42 °C, and 5 μl of the first-strand RT product was used for PCR amplification. PCR conditions for endogenous *Pax7* were 94 °C for 5 min; 35 cycles of 94 °C for 45 s, 56 °C for 45 s, 72 °C for 45 s; and finally 72 °C for 7 min. The PCR primers span intron 8 of the *Pax7* gene (Pax7-exon8-fwd 5′ gct acc agt aca gcc agt atg 3′ and Pax7-exon9-rev 5′ gtc act aag cat ggg tag atg 3′) and amplify sequence in the 3′-UTR of the gene that is not contained in the viral Pax7 expression cassette. RT-PCR products were analyzed by electrophoresis through a TAE-ethidium-agarose gel.

Northern blot studies were performed according to standard techniques using random-primed ^32^P-dCTP radiolabeled cDNA fragments as probes (Redi-prime, Amersham Biosciences)(Sabourin et al. 1999). Fifteen micrograms of total RNA from various cell cultures was electrophoresed in denaturing formaldehyde gels and transferred to Hybond-N filters (Amersham Biosciences).

## Supporting Information

### Accession Numbers

The accession numbers for the proteins discussed in this paper are Desmin (LocusLink ID 13346), mouse MyoD (GenBank NM_010866), MyoD (LocusLink ID 17927), Myogenin (LocusLink ID 17928), Pax7 (LocusLink ID 18509), and Pax7d isoform (GenBank AF_254422).

## Abbreviations

Ad-Pax7: adenovirus-Pax7
Ad-LacZ: adenovirus-*β-galactosidase;* CDSC-Pax7
ctx: cardiotoxin
DSHB: Developmental Studies Hybridoma Bank
HAN-puro: retroviral HAN-puromycin
MRF: myogenic regulatory factor
MyHC: myosin heavy chain
RT-PCR: reverse transcriptase PCR
SP: side population
TA: tibialis anterior

## References

[pbio-0020130-Asakura1] Asakura A, Seale P, Girgis-Gabardo A, Rudnicki MA (2002). Myogenic specification of side population cells in skeletal muscle. J Cell Biol.

[pbio-0020130-Bendall1] Bendall AJ, Ding J, Hu G, Shen MM, Abate-Shen C (1999). Msx1 antagonizes the myogenic activity of Pax3 in migrating limb muscle precursors. Development.

[pbio-0020130-Bischoff1] Bischoff R (1994). The satellite cell and muscle regeneration. In: Myogenesis, AG Engel, C Franszini-Armstrong, editors.

[pbio-0020130-Blaveri1] Blaveri K, Heslop L, Yu DS, Rosenblatt JD, Gross JG (1999). Patterns of repair of dystrophic mouse muscle: Studies on isolated fibers. Dev Dyn.

[pbio-0020130-Borycki1] Borycki AG, Li J, Jin F, Emerson CP, Epstein JA (1999). Pax3 functions in cell survival and in pax7 regulation. Development.

[pbio-0020130-Bulfield1] Bulfield G, Siller WG, Wight PA, Moore KJ (1984). X chromosome–linked muscular dystrophy (mdx) in the mouse. Proc Natl Acad Sci U S A.

[pbio-0020130-Cao1] Cao B, Zheng B, Jankowski RJ, Kimura S, Ikezawa M (2003). Muscle stem cells differentiate into haematopoietic lineages but retain myogenic potential. Nat Cell Biol.

[pbio-0020130-Charge1] Charge SBP, Rudnicki MA (2004). Cellular and molecular regulation of muscle regeneration. Physiol Rev.

[pbio-0020130-Cossu1] Cossu G, Kelly R, Tajbakhsh S, Di Donna S, Vivarelli E (1996). Activation of different myogenic pathways: Myf-5 is induced by the neural tube and MyoD by the dorsal ectoderm in mouse paraxial mesoderm. Development.

[pbio-0020130-Delfini1] Delfini M, Hirsinger E, Pourquie O, Duprez D (2000). Delta 1–activated notch inhibits muscle differentiation without affecting Myf5 and Pax3 expression in chick limb myogenesis. Development.

[pbio-0020130-Goulding1] Goulding M, Lumsden A, Paquette AJ (1994). Regulation of Pax-3 expression in the dermomyotome and its role in muscle development. Development.

[pbio-0020130-Graham1] Graham FL, van der Eb AJ (1973). Transformation of rat cells by DNA of human adenovirus 5. Virology.

[pbio-0020130-Gussoni1] Gussoni E, Soneoka Y, Strickland CD, Buzney EA, Khan MK (1999). Dystrophin expression in the mdx mouse restored by stem cell transplantation. Nature.

[pbio-0020130-Heanue1] Heanue TA, Reshef R, Davis RJ, Mardon G, Oliver G (1999). Synergistic regulation of vertebrate muscle development by Dach2, Eya2, and Six1, homologs of genes required for *Drosophila* eye formation. Genes Dev.

[pbio-0020130-Heslop1] Heslop L, Morgan JE, Partridge TA (2000). Evidence for a myogenic stem cell that is exhausted in dystrophic muscle. J Cell Sci.

[pbio-0020130-Jackson1] Jackson KA, Mi T, Goodell MA (1999). Hematopoietic potential of stem cells isolated from murine skeletal muscle. Proc Natl Acad Sci U S A.

[pbio-0020130-Kay1] Kay PH, Mitchell CA, Akkari A, Papadimitriou JM (1995). Association of an unusual form of a Pax7-like gene with increased efficiency of skeletal muscle regeneration. Gene.

[pbio-0020130-Mansouri1] Mansouri A, Stoykova A, Torres M, Gruss P (1996). Dysgenesis of cephalic neural crest derivatives in *Pax7*
**^ −/−^** mutant mice. Development.

[pbio-0020130-Maroto1] Maroto M, Reshef R, Munsterberg AE, Koester S, Goulding M (1997). Ectopic Pax-3 activates MyoD and Myf-5 expression in embryonic mesoderm and neural tissue. Cell.

[pbio-0020130-McKinney-Freeman1] McKinney-Freeman SL, Jackson KA, Camargo FD, Ferrari G, Mavilio F (2002). Muscle-derived hematopoietic stem cells are hematopoietic in origin. Proc Natl Acad Sci U S A.

[pbio-0020130-Megeney1] Megeney LA, Kablar B, Garrett K, Anderson JE, Rudnicki MA (1996). MyoD is required for myogenic stem cell function in adult skeletal muscle. Genes Dev.

[pbio-0020130-Montarras1] Montarras D, Lindon C, Pinset C, Domeyne P (2000). Cultured myf5 null and myoD null muscle precursor cells display distinct growth defects. Biol Cell.

[pbio-0020130-Munsterberg1] Munsterberg AE, Lassar AB (1995). Combinatorial signals from the neural tube, floor plate and notochord induce myogenic bHLH gene expression in the somite. Development.

[pbio-0020130-Pagel1] Pagel CN, Partridge TA (1999). Covert persistence of mdx mouse myopathy is revealed by acute and chronic effects of irradiation. J Neurol Sci.

[pbio-0020130-Parker1] Parker MH, Seale P, Rudnicki MA (2003). Looking back to the embryo: Defining transcriptional networks in adult myogenesis. Nat Rev Genet.

[pbio-0020130-Petropoulos1] Petropoulos H, Skerjanc IS (2002). Beta-catenin is essential and sufficient for skeletal myogenesis in p19 cells. J Biol Chem.

[pbio-0020130-Polesskaya1] Polesskaya A, Seale P, Rudnicki MA (2003). Wnt Signaling Induces the Myogenic Specification of Resident CD45+ Adult Stem Cells during Muscle Regeneration. Cell.

[pbio-0020130-Pourquie1] Pourquie O, Coltey M, Breant C, Le Douarin NM (1995). Control of somite patterning by signals from the lateral plate. Proc Natl Acad Sci U S A.

[pbio-0020130-Pourquie2] Pourquie O, Fan CM, Coltey M, Hirsinger E, Watanabe Y (1996). Lateral and axial signals involved in avian somite patterning: A role for BMP4. Cell.

[pbio-0020130-Qu-Petersen1] Qu-Petersen Z, Deasy B, Jankowski R, Ikezawa M, Cummins J (2002). Identification of a novel population of muscle stem cells in mice: Potential for muscle regeneration. J Cell Biol.

[pbio-0020130-Sabourin1] Sabourin LA, Girgis-Gabardo A, Seale P, Asakura A, Rudnicki MA (1999). Reduced differentiation potential of primary MyoD−/− myogenic cells derived from adult skeletal muscle. J Cell Biol.

[pbio-0020130-Schultz1] Schultz E, Jaryszak DL, Gibson MC, Albright DJ (1986). Absence of exogenous satellite cell contribution to regeneration of frozen skeletal muscle. J Muscle Res Cell Motil.

[pbio-0020130-Seale1] Seale P, Sabourin LA, Girgis-Gabardo A, Mansouri A, Gruss P (2000). Pax7 is required for the specification of myogenic satellite cells. Cell.

[pbio-0020130-Sicinski1] Sicinski P, Geng Y, Ryder-Cook AS, Barnard EA, Darlison MG (1989). The molecular basis of muscular dystrophy in the mdx mouse: A point mutation. Science.

[pbio-0020130-Soneoka1] Soneoka Y, Cannon PM, Ramsdale EE, Griffiths JC, Romano G (1995). A transient three-plasmid expression system for the production of high titer retroviral vectors. Nucleic Acids Res.

[pbio-0020130-Tajbakhsh1] Tajbakhsh S, Bober E, Babinet C, Pournin S, Arnold H (1996). Gene targeting the myf-5 locus with nlacZ reveals expression of this myogenic factor in mature skeletal muscle fibres as well as early embryonic muscle. Dev Dyn.

[pbio-0020130-Tajbakhsh2] Tajbakhsh S, Rocancourt D, Cossu G, Buckingham M (1997). Redefining the genetic hierarchies controlling skeletal myogenesis: Pax- 3 and Myf-5 act upstream of MyoD. Cell.

[pbio-0020130-Tajbakhsh3] Tajbakhsh S, Borello U, Vivarelli E, Kelly R, Papkoff J (1998). Differential activation of Myf5 and MyoD by different Wnts in explants of mouse paraxial mesoderm and the later activation of myogenesis in the absence of Myf5. Development.

[pbio-0020130-Torrente1] Torrente Y, Tremblay JP, Pisati F, Belicchi M, Rossi B (2001). Intraarterial injection of muscle-derived CD34(+)Sca-1(+) stem cells restores dystrophin in mdx mice. J Cell Biol.

[pbio-0020130-Wakeford1] Wakeford S, Watt DJ, Partridge TA (1991). X-irradiation improves mdx mouse muscle as a model of myofiber loss in DMD. Muscle Nerve.

[pbio-0020130-Williams1] Williams BA, Ordahl CP (1994). Pax-3 expression in segmental mesoderm marks early stages in myogenic cell specification. Development.

[pbio-0020130-Ziman1] Ziman MR, Fletcher S, Kay PH (1997). Alternate Pax7 transcripts are expressed specifically in skeletal muscle, brain and other organs of adult mice. Int J Biochem Cell Biol.

